# The Effect of *Lycium Barbarum* Polysaccharide on Alcohol-Induced Oxidative Stress in Rats

**DOI:** 10.3390/molecules16032542

**Published:** 2011-03-17

**Authors:** Daye Cheng, Hong Kong

**Affiliations:** 1 Department of Transfusion, The First Hospital of China Medical University, Shenyang, Liaoning Province, China; 2 Department of Laboratory Medicine, Shengjing Hospital of China Medical University, Shenyang, Liaoning Province, China; E-Mail: konghong_cmu@yahoo.cn

**Keywords:** *Lycium barbarum* Polysaccharide, alcoholic liver disease, oxidative stress

## Abstract

The aim of this study was to investigate the effects of *Lycium barbarum* Polysaccharide (LBP) on alcohol-induced liver damage in rats. A total of 36 rats were divided into control, ethanol and ethanol + LBP groups. Rats in the ethanol group were fed 7 g ethanol/kg body weight by gastric infusion, three times a day, for 30 consecutive days, while rats in the control group received the same volume of physiological saline instead of ethanol, and rats in ethanol + LBP group were fed both ethanol (7 g/kg body weight) and LBP (300 mg/Kg body weight/day). Alcoholic liver injury was examined by serum ALT and AST activities, alcoholic fatty liver was assessed by lipid levels, and oxidative stress was evaluated by SOD, CAT, GSH-Px, GSH and MDA assays. In the ethanol group, a significant elevation of enzymes and lipid in serum, increased MDA level and depletion of SOD, CAT, GSH-Px and GSH in liver were observed. LBP administration significantly ameliorated liver injury, prevented the progression of alcohol-induced fatty liver, and improved the antioxidant functions when compared with the ethanol group. Histopathological examination of rat liver revealed that LBP administration protected liver cells from the damage induced by ethanol. The results suggest that LBP is a promising agent to protect the liver from hepatotoxicity and fatty liver induced by ethanol intake.

## 1. Introduction 

According to a WHO 2008 report, alcohol causes 1.8 million annual deaths globally and accounts for 4.0% of the total disease burden [1]. Alcoholic liver disease (ALD) is a complex multistep chronic disease process which typically progresses through the stages of alcoholic steatosis, alcoholic hepatitis and alcoholic cirrhosis to end-stage liver disease [2]. Numerous data on the pathogenesis of ALD have been obtained from animal studies [3,4,5]. Chronic alcohol consumption induces hepatic oxidative stress due to increased generation of reactive oxygen species and/or reduced antioxidant capacity [6]. Oxidative stress causes further lipid peroxidation, which can directly damage the membranes of cells and organelles and lead to the release of reactive aldehydes with potent pro-inflammatory and pro-fibrotic properties [7]. Thus, alternative health care practitioners routinely recommend natural antioxidant supplements for ALD. 

Considerable interest has developed over the last decades in plants like *Lycium barbarum* (family Solanaceae; commercially known as goji berry), which is well-known in traditional Chinese herbal medicine and has been widely used as a popular functional food with a large variety of beneficial effects, such as lowering blood glucose and serum lipid, anti-aging, immuno-modulating, antitumor, anti-fatigue, and male fertility-facilitating [8,9,10,11,12]. The bioactivity of *Lycium barbarun* is mainly attributed to its polysaccharide complex, which contains several fractions separable by ion exchange chromatography. *Lycium barbarum Polysaccharide* (LBP) generally consists of six monosaccharides (galactose, glucose, rhamnose, arabinose, mannose, and xylose). 

The purpose of this study was to investigate whether LBP administration could prevent alcohol-induced oxidative stress. To this end serum transaminase activities, lipid levels, enzymatic and nonenzymatic antioxidants along with histopathological changes in the liver, were observed and evaluated in three groups of rats treated by alcohol with and without LBP, plus a control group. A previous report indicated that LBP can protect against liver damage induced by CCl_4_ [13].

## 2. Results and Discussion 

### 2.1. Chemical components and structural analysis

The characteristic absorptions of LBP were identified in the FT-IR spectrum, where carbohydrate related absorbances appear at 3,400.38, 2,930.49, 1,629.66, 1,411.40, 1,151.44, 1,078.24, 1,032.50, 920.72, 864.33, 817.08, and 777.04 cm^−1^. The signal at 3,400.38–2,930.49 cm^−1^ might correspond to the bending vibration of C–H, C=C bonds. The absorbance at 1,629.66 cm^−1^ can be assigned to CH_3_–, CH_2_– groups. The small sharp band at 920 cm^−1^ is characteristic of β-glycosidic linkages between the sugars, indicating polysaccharides joined by this type of link. 

### 2.2. Effect of LBP on body weight (BW) in experiment rats

In the present study, effect of LBP administration on body weight of rats was examined. During the initial day of feeding, there was no significant difference in body weights in the three groups (*p* > 0.05). After the 30-day experiment, no significant difference was found between the three groups (*p* > 0.05), as shown in Table 1.

### 2.3. Effects of LBP on liver damage in experiment rats

**Figure 1:**
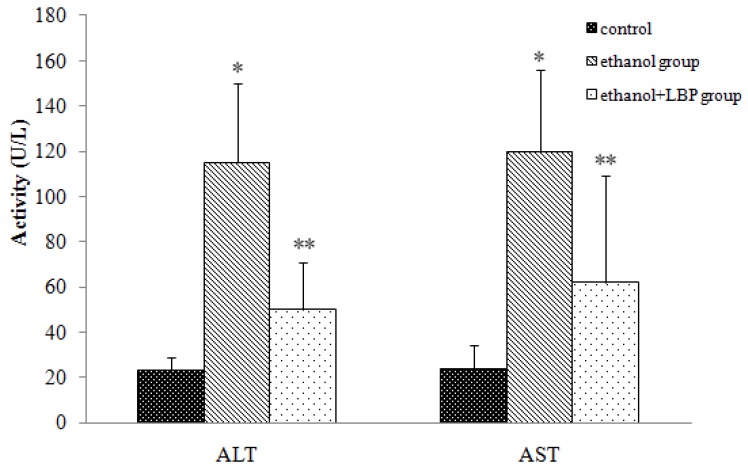
Effects of LBP on serum ALT and AST activities in rats. Values are expressed as mean ± SD for 12 rats in each group. * *p* < 0.01, ethanol group *vs.* control group. ** *p* < 0.01, ethanol + LBP group *vs.* ethanol group.

The most prominent result of liver damage is the release of intracellular enzymes such as alanine aminotransferase (ALT) and aspartate aminotransferase (AST) into the blood stream, which leads to an increase in serum levels of those enzymes, therefore serum ALT and AST levels can serve as indicators of liver status, e.g. higher values might reflect the liver damage. As shown in Figure 1, serum ALT and AST activities in the ethanol group increased significantly (*p* < 0.01) in comparison with those of the control group, but serum ALT and AST activities in the ethanol + LBP group were significantly (*p* < 0.01) lower than in the ethanol group, suggesting that LBP is helpful in preventing liver damage.

### 2.4. Effects of LBP on serum lipid level in experiment rats

**Figure 2:**
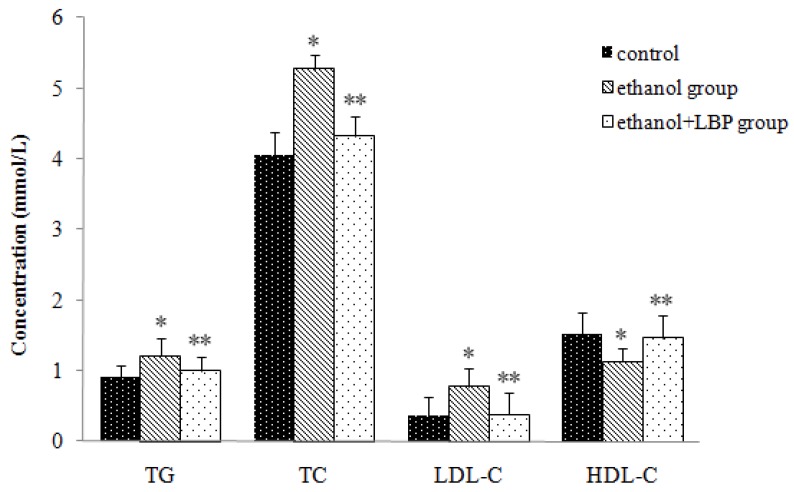
Effects of LBP on serum lipid levels in rats. Values are expressed as mean ± SD for 12 rats in each group. * *p* < 0.01, ethanol group *vs.* control group. ** *p* < 0.01, ethanol + LBP group *vs.* ethanol group.

Figure 2 shows the changes of serum triglycerides (TG), total cholesterol (TC), high density lipoprotein cholesterol (HDL-C), and low density lipoprotein cholesterol (LDL-C) in the different experimental groups. The ethanol group displayed significant increases in the levels of serum TG, TC and LDL-C (*p* < 0.01) and a decrease in the level of serum HDL-C (*p* < 0.01) in comparison with the control group. In the ethanol + LBP group, serum TG, TC, and LDL-C levels decreased remarkably (*p* < 0.01), whereas the HDL-C level increased significantly (*p* < 0.01) compared with the ethanol group. Antioxidants such as LBP have been reported to reduce high fat-induced liver damage [14]. Similarly, we observed that LBP administration significantly reduced lipid accumulation. This suggests that LBP might be helpful to repair a damaged lipid metabolism or reverse the lipid dysfunction caused by ethanol administration in rats and prevent the progression of alcohol-induced fatty liver.

### 2.5. Effects of LBP on antioxidant levels and lipid peroxidation levels in rat liver

Table 2 depicts the levels of antioxidant enzymes like superoxide dismutase (SOD), catalase (CAT), glutathione-dependent peroxidase (GSH-Px), the level of a non-enzymatic antioxidant like glutathione (GSH) and a lipid peroxidation product like malondialdehyde (MDA) in rat livers in the different experimental groups. 

Antioxidant enzyme activities and GSH level in the ethanol group decreased significantly (*p* < 0.01), whereas MDA levels increased compared with the control group (*p* < 0.01). LBP administration significantly (*p* < 0.01) blocked the decreases in antioxidant levels (SOD, GPx, CAT and GSH) and the increase in MDA level (*p* < 0.01) in the alcohol-fed rats. SOD, CAT, and GSH-Px are all antioxidant enzymes that act cooperatively at different sites in the metabolic pathways involving free radicals. The end products of lipid peroxidation were assessed through the level of MDA, a known biomarker of lipid peroxidation and oxidative stress. The decrease of MDA levels in ethanol + LBP treated rats may be partially due to a conteraction of the deleterious effects of lipid peroxidation by LBP utilization, suggesting that LBP plays an important role in repairing of lipid dysfunction led by alcohol-induced liver damage. From a toxicological point of view, the importance of GSH in the detoxification of chemically reactive metabolites has also been extensively documented, with numerous examples of drug-induced toxicity after GSH depletion [15,16]. GSH decrease may due to increased oxidation of GSH or decreased synthesis of GSH and/or decreased availability of precursors for GSH formation. Thus it is reasonable to think of increasing of GSH and other antioxidant enzymes such as SOD, CAT and GSH-Px might contribute to antagonize the oxidative stress effects. Our results strongly indicate that LBP can improve the antioxidant functions either by scavenging excessive alcohol-induced free radicals or by promoting antioxidant enzyme activities and can be used as therapeutic agent to alcohol-induced liver damage.

### 2.6. Histopathological analysis

**Figure 3:**
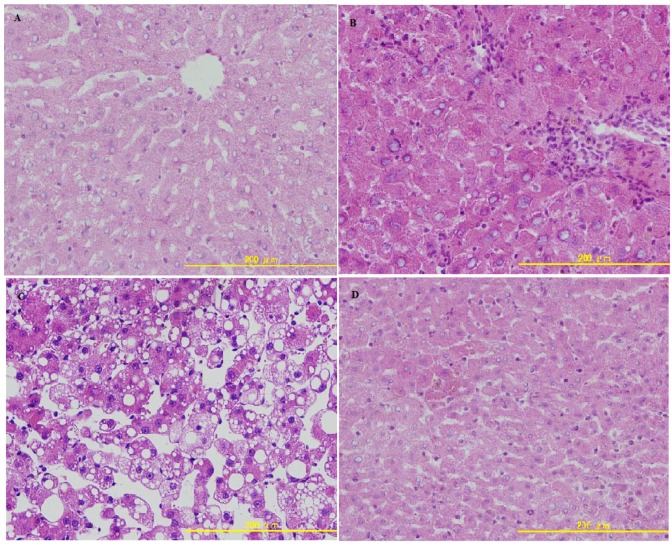
(a) Photomicrograph of a section of the liver of a normal rat, showing the architecture of the hepatic lobule. (b) Photomicrograph of a section of the liver of an alcohol-induced rat showing lymphocytic infiltration and narrowness of central vein resembling chronic hepatitis. (c) Photomicrograph of a section of the liver of an alcohol-induced rat showing necrosis and fatty changes. (d) Photomicrograph of a section of the liver of rat treated with LBP showing nearly the same appearance as the control.

Liver biopsies are useful to evaluate the stage and severity of ALD. As shown in Figure 3, in the control group, liver sections had normal hepatic cell with preserved cytoplasm, distinct nucleus and central vein. 

Livers of ethanol treated rats displayed lymphocytic infiltration and central vein narrowness resembling chronic hepatitis. In addition, the presence of massive fatty degeneration, inflammatory cell infiltration and loss of cellular boundaries were also noted in the ethanol group. Rats in the ethanol + LBP group exhibited marked improvements in their liver histopathology, suggesting that LBP is capable of not only preventing, but actually reversing the pathomorphological changes of ALD, such as changes in fat deposition and inflammatory cell infiltration.

## 3. Experimental

### 3.1. Materials

Fruits of *Lycium barbarum* were purchased from the Shenyang Company (People’s Republic of China). All reagents used in this research were of analytical grade and obtained from the Shenyang Biotechnology Co. Ltd.

### 3.2. Extraction of polysaccharides

Dried fruits of *Lycium barbarum* (200 g) were defatted in a Soxhlet 6 L apparatus with petroleum ether (300 mL, boiling point: 60–90 °C). The residue obtained from the petroleum ether extraction was treated twice with 80% ethanol (100 mL) to remove some coloured materials, monosaccharides, oligosaccharides, and small molecule materials. The organic solvent was volatilized and a pretreated dry powder was obtained that was immersed in distilled water for 2 h. The suspensions were extracted with water under reflux for 4 h. The water phase was filtered and freeze-dried. All the polysaccharides were stored at −20 °C.

### 3.3. Preparation of Lyceum barbarum Polysaccharides 

The crude polysaccharide was further purified by column chromatography. Fifty milligrams of crude polysaccharide dissolved in dH_2_O (10 mL) was applied to a DEAE–cellulose column (3 cm × 45 cm) preequilibrated with water and eluted in a NaCl gradient (0–3 M) until no carbohydrate was detected. Each fraction was assayed for carbohydrate content by the phenol-sulfuric acid method [17]. The carbohydrate-positive fractions were pooled together and dialyzed (MWCO 14,000) for 24 h against dH_2_O and then lyophilized. Seven small fractions and one major fraction were observed. The major fraction was taken as purified LBP and used in infrared analysis and animal tests.

### 3.4. Infrared spectral analysis

The IR spectrum was recorded by a Nicolet Magna 750 FT-IR spectrophotometer in the range of 4,000–400 cm^−1^ as KBr pellets.

### 3.5. Animals

A total of 36 male rats (200–220 g) averaging 12 weeks old were obtained from the Animal Center of China Medical University. Rats were fed with a standard commercial laboratory chow and had free access to water. The rats were housed in wire-bottomed stainless cages in a temperature controlled room (23 ± 1 °C) and maintained at a 12 h light/dark photoperiod. After a 5-day adaption period, the rats were divided into three groups (*n* = 12): control group, ethanol group, and ethanol + LBP group. Ethanol group rats were given ethanol diluted with normal saline (56%; v/v) administered by gastric infusion at a dose of 7 g/kg body weight, three times a day, for 30 consecutive days (binge alcohol consumption). Control group rats were fed with the same volume of physiological saline by gastric infusion for 30 consecutive days; Ethanol + LBP group rats were fed LBP (300 mg/Kg body weight/day) in drinking water administered for 30 consecutive days, and ethanol was given simultaneously as described for the ethanol group. Eight hours after the administration of the last ethanol doses, rats were euthanized. In the experiments the body weights of the rats were recorded initially, and at the end of the experiment. Blood samples were collected and centrifuged at 1,500 g/min at 4 °C for 10 min to obtain serum. Livers were totally excised from the rats and stored at −80 °C for the subsequent experiments.

### 3.6. Determinations

#### 3.6.1. Measurement of ALT and AST in serum 

The degree of liver damage was evaluated by ALT and AST in serum using a commercially available kit (Abbott Company, USA) by HITACHI 7170 autoanalyzer according to the manufacturer’s instructions. 

#### 3.6.2. Determination of lipid levels in serum

Serum TG, TC, and HDL-C were analyzed by the enzymatic reaction method on a HITACHI 7170 autoanalyzer using a commercially available kit (KYOMA, Japan). Serum LDL-C was calculated using the Friedewald equation.

#### 3.6.3. Oxidative Stress parameters in rat livers

SOD activity in liver was determined according to the method described by Marklund and Marklund [18]. GSH-Px activity was determined by GSH-Px assay kit (Nanjing Jiancheng Biology Research Institute, Nanjing, P.R. China). CAT was assayed by the method described by Ferro Cde *et al*. [19]. The non-enzymic GSH was analyzed by the method of Moron, Dipierre, and Mannervik [20]. Lipid peroxidation was evaluated on the base of MDA level [21], and MDA in liver was determined using the method described by Jain *et al.* [22].

### 3.7. Histopathological studies

Rat livers from all groups were removed and fixed immediately in 10% neutral buffered formalin, dehydrated in gradual ethanol (50%–100%), cleared in xylene and embedded in paraffin. Sections (4–5 μm thick) were prepared and stained with hematoxilen and eosin (H&E) for photomicroscopic observation.

### 3.8. Statistical analyses

All data were expressed as mean ± S.D. Differences between groups were assessed by analysis of variance and *t*-test. A value of *p* < 0.05 was considered to be statistically significant. All statistical analyses were carried out using SPSS 15.0 software (SPSS, Chicago, IL, USA).

## 4. Conclusions

In this study, LBP administration significantly inhibited the increase of serum ALT and AST activities caused by ethanol intake. In addition, LBP administration significantly prevented the progression of alcohol-induced fatty liver. Furthermore, LBP administration not only elevated the antioxidants levels (GSH, SOD, CAT and GPx), but also reduced the lipid peroxidation product (MDA) level. These results clearly demonstrated that LBP is a promising agent to protect the liver from hepatotoxicity and fatty liver induced by ethanol intake. These results have shed some light on the clinical therapeutic potential of LBP against alcohol-induced liver damage. 

## Figures and Tables

**Table 1:** Effect of LBP on body weights of rats in three groups.

	Control group	Ethanol group	Ethanol+LBP group
**Initial body weight (g)**	209.3 ± 4.1	209.5 ± 5.8	211.3 ± 5.4
**Final body weight (g)**	237.5 ± 7.7	236.3 ± 5.9	239.8 ± 6.9

Note: Data represent mean ± SD from 12 rats in each group.

**Table 2:** Effect of LBP on oxidative stress parameters in rat livers.

	MDA (nmol/mg protein)	SOD (U/mg protein)	CAT (U/mg protein)	GPx (U/mg protein)	GSH (μg/mg protein)
**Control group**	7.27 ± 0.76	12.53 ± 4.47	19.88 ± 3.79	4.03 ± 0.87	7.49 ± 0.89
**Ethanol group**	10.19 ± 1.36 ^*^	6.98 ± 2.85 ^*^	12.79 ± 3.17^*^	1.46 ± 0.58 ^*^	5.10 ± 0.64 ^*^
**Ethanol+LBP group**	7.47 ± 0.90 ^**^	12.07 ± 4.15 ^**^	17.06 ± 3.73 ^**^	3.50 ± 0.70 ^**^	6.57 ± 0.54 ^**^

Note: Data represent mean ± SD from 12 rats in each group. ^*^ *p* < 0.01, ethanol group *vs.* control group; ^**^ *p* < 0.01, ethanol + LBP group *vs.* ethanol group.
